# Supplementary material for: Reducing greenhouse gas emissions of Amazon hydropower with strategic dam planning
Source: Nat Commun. 2019 Sep 19;10:4281. doi: 10.1038/s41467-019-12179-5 (PMC6753097; doi:10.1038/s41467-019-12179-5)
Supplement: Supplementary file 1 — Supplementary Information [file 41467_2019_12179_MOESM1_ESM.pdf]

Supplementary Information for

Reducing greenhouse gas emissions of Amazon hydropower with strategic dam planning

Rafael M. Almeida*, Qinru Shi, Jonathan M. Gomes-Selman, Xiaojian Wu, Yexiang Xue, Hector Angarita, Nathan Barros, Bruce R. Forsberg, Roosevelt García-Villacorta, Stephen K. Hamilton, John M. Melack, Mariana Montoya, Guillaume Perez, Suresh A. Sethi, Carla P. Gomes, Alexander S. Flecker

*Corresponding authors, rafaelmalmeida2@gmail.com, gomes@cs.cornell.edu and asf3@cornell.edu

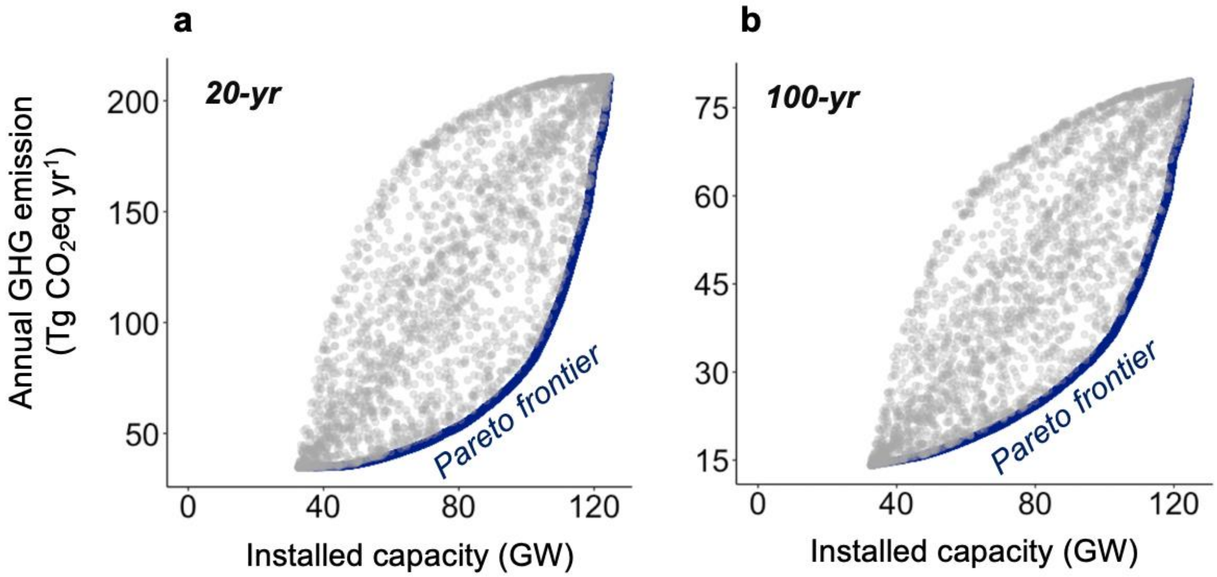

Supplementary Figure 1. **Tradeoffs between hydropower generation and annual greenhouse gas emission for portfolios of proposed Amazon dams**. Each point represents a portfolio of dams from among 351 proposed projects in the Amazon. The optimal dam portfolios for each value of installed capacity (Pareto frontier) are shown in dark blue, and randomly generated suboptimal dam portfolios are shown by grey symbols. Results are shown starting from existing installed capacity for the set of 158 existing dams (33 GW for an emission of 34 Tg CO_2eq yr^{-1} over a 20-year horizon and 11 Tg CO_2eq yr^{-1} over a 100-year horizon) and presented over a (**a**) 20- and (**b**) 100-year horizon.1 Tg = 10^{12} g.

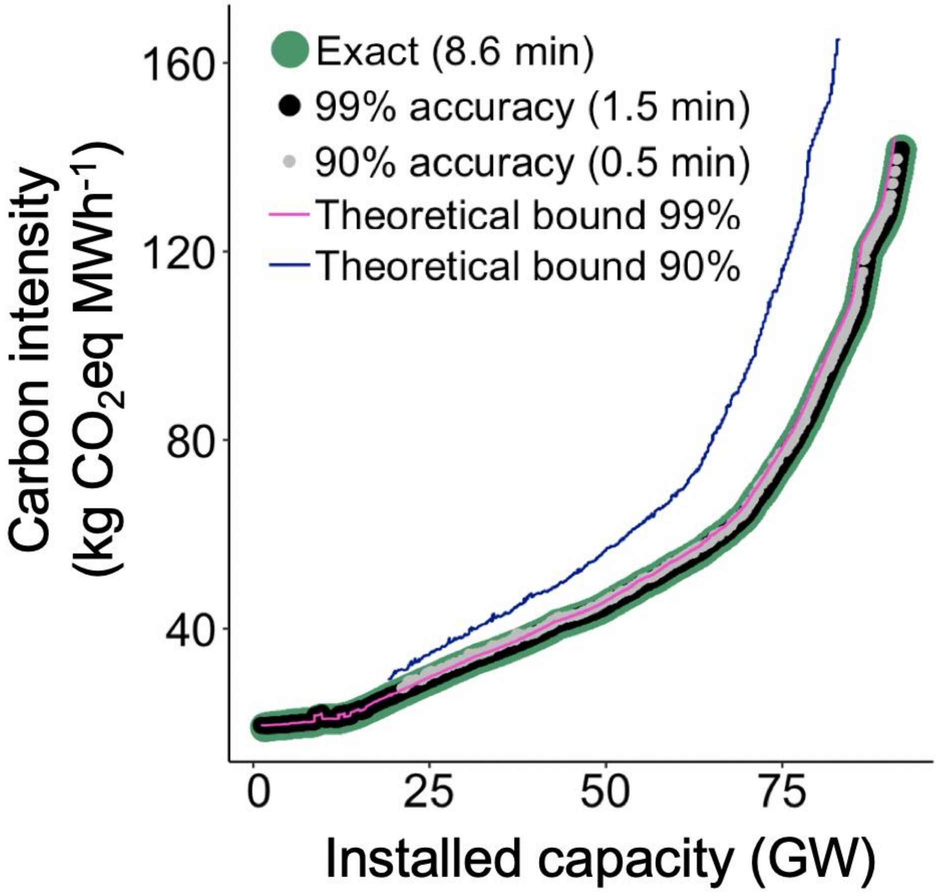

Supplementary Figure 2. **Exact and approximate Pareto optimal frontiers for proposed dams.** The Pareto frontiers indicate the tradeoff analysis between installed capacity and carbon intensity (100-year time horizon) for the set of 351 proposed dams. The approximate frontiers were computed with 99% and 90% accuracies ($\varepsilon = 0.01$ and 0.1, respectively). While the computation of the exact (or provably optimal) Pareto frontier (green points) takes only around 10 minutes, computation of the approximate Pareto frontiers was much faster (1.5 minutes for an accuracy of 99%, black points; 0.5 minutes for an accuracy of 90%, gray points). Also included are lines indicating the theoretical guarantees for the approximate solutions. The actual accuracies of the approximate solutions are much higher than their theoretical guarantees. The approximate Pareto frontier computed with a guarantee of 90% accuracy is only 1% away from the exact Pareto frontier, in addition to overlapping with the 99% theoretical guarantee.

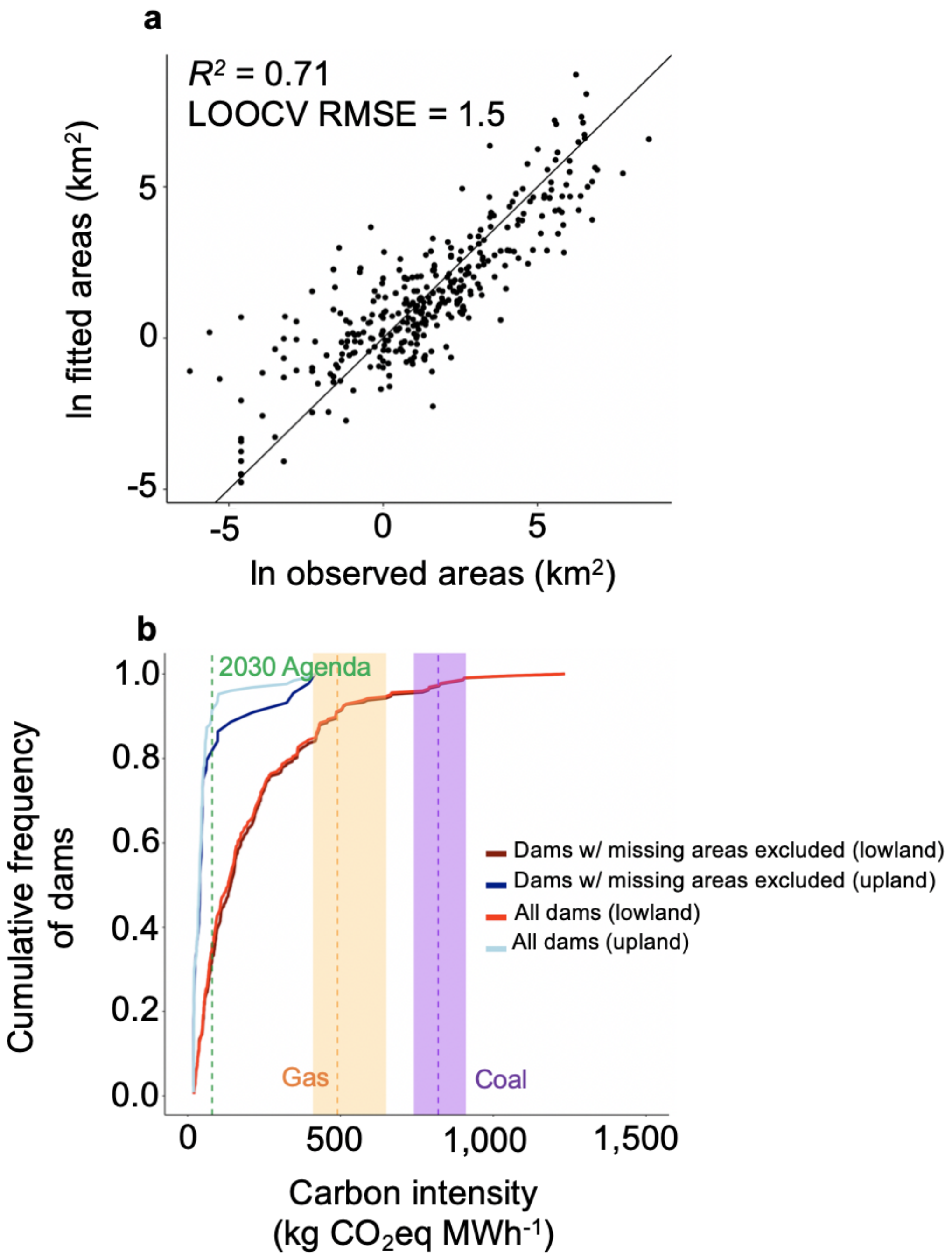

Supplementary Figure 3. Estimating missing flooded areas. Flooded areas for the subset of dams with missing information were estimated using normal multiple linear regression. We regressed natural log of flooded area (km^2) against a country categorical covariate (levels = Bolivia, Brazil, Ecuador, Peru), natural log of installed capacity (MW), natural log of dam watershed area (km^2), and natural log of elevation (m a.s.l.) using a set of n = 333 existing or

proposed dams for which reported flooded areas were available. **a,** Regression model performance displaying the observed flooded areas against fitted areas, the coefficient of determination (R^2) for the fitted normal multiple linear regression model, and root mean squared error (RMSE) predictive accuracy based on leave-one-out cross validation (LOOCV). **b,** Sensitivity analysis demonstrates robustness of conclusions to the use of estimated flooded areas, showing that the cumulative frequency distribution of lowland and upland dam carbon intensities (100-year time horizon) are similar when the subset of dams with estimated flooded areas are included or excluded from analyses, with the exception of upland dams with high carbon intensities.

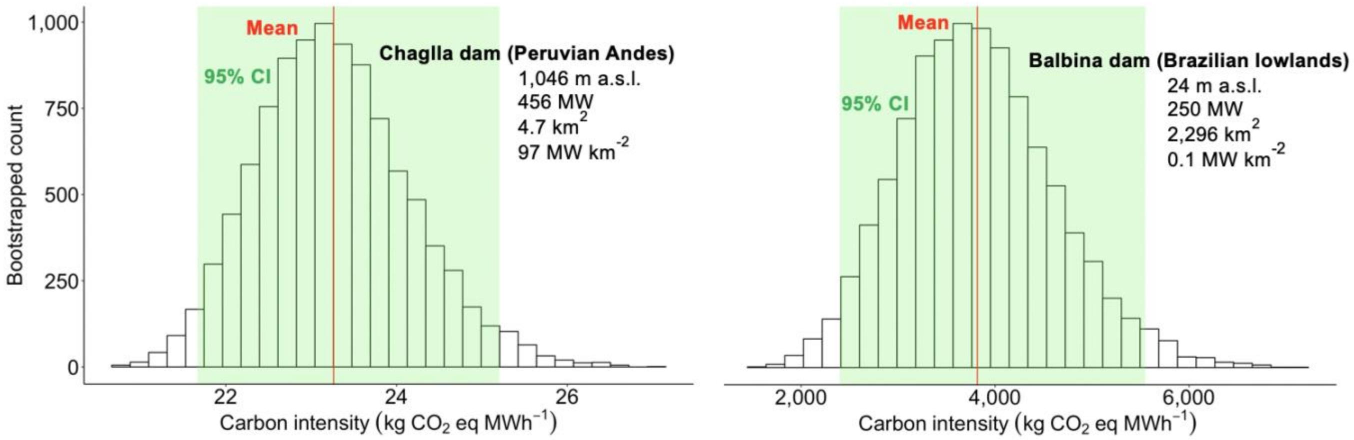

Supplementary Figure 4. Examples of the bootstrapping output for existing Amazon dams with contrasting power densities. Histograms indicating the 10,000 carbon intensities resulting from the bootstrapping output for Chaglla dam in Peru (left) and Balbina dam in Brazil (right), considering a 100-year time horizon. The elevation above sea level, installed capacity, flooded area, and power density of the dams are indicated in the graphs. The calculated carbon intensity of a dam with the power density of Chaglla is below that of a solar power plant[7] (~ 50 kg CO_2eq MWh^{-1}) with equivalent power generation even in a worst-case scenario for emissions. Conversely, a dam such as Balbina would emit more than a coal-fired power plant (~ 800 kg CO_2eq MWh^{-1}) even in a best-case scenario for emissions. Indeed, previous work indicated that Balbina emits more GHGs than any type of conventional fossil fuel-fired power plant with equivalent energy production[11].

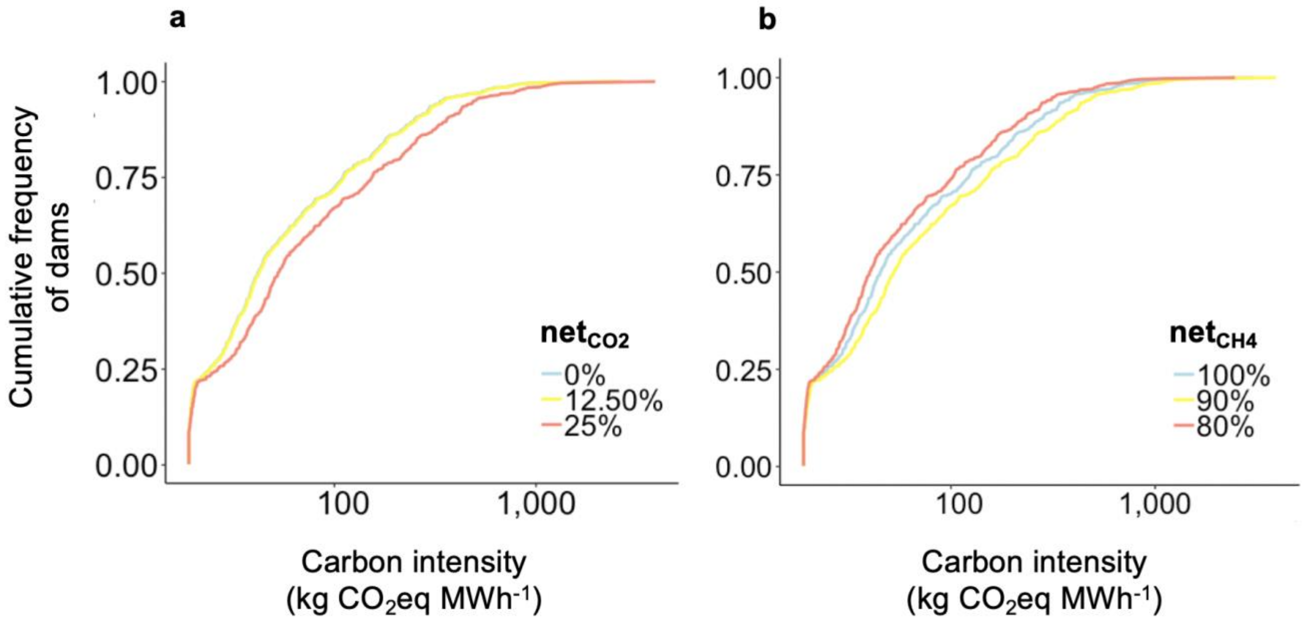

Supplementary Figure 5. Sensitivity of estimated carbon intensities to variation in the assumed net CO_2 and CH_4 emissions due to reservoir creation. Our analysis considers that net CO_2 and CH_4 emissions are equivalent to 25% and 90% of gross emissions, respectively, because there would have been natural emissions of these gases prior to impoundment. We varied these percentages separately for CO_2 and CH_4 to examine the sensitivity of the resultant frequency distributions to those assumed percentages. **a,** for CO_2, modeled carbon intensities decrease by ≈ 20% when we assume that all CO_2 emissions would have occurred in pre-impoundment conditions (i.e., 0% parameter). **b,** for CH_4, modeled carbon intensities decrease by ≈ 30% when we assume that 20% of CH_4 emissions would occur in pre-impoundment conditions (i.e., 80% parameter). These sensitivity analyses were run for the 100-year time horizon estimates.

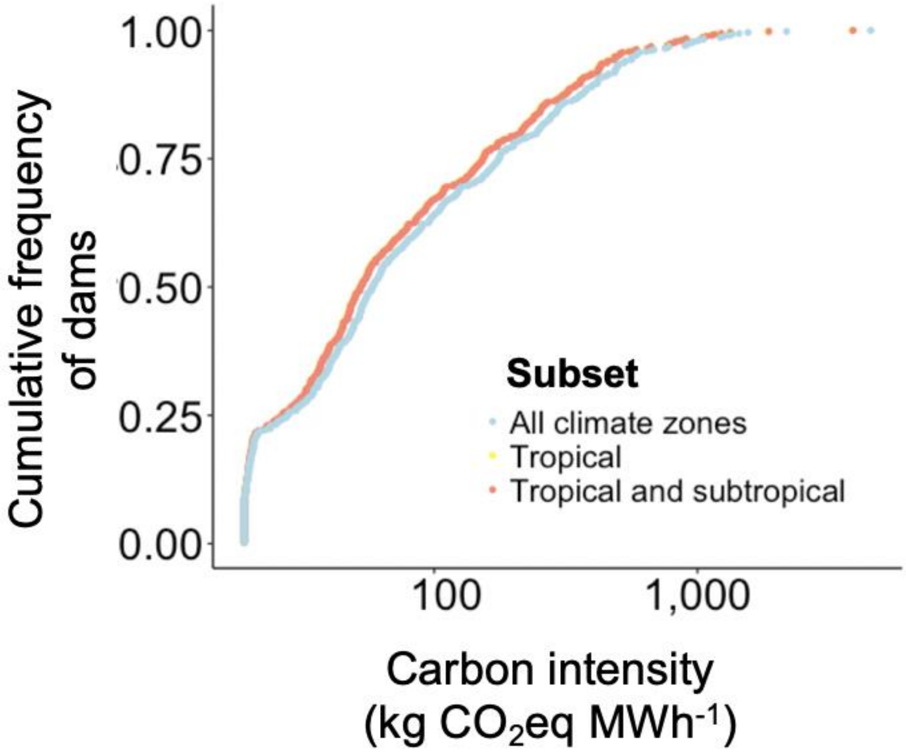

Supplementary Figure 6. Sensitivity of estimated carbon intensities to variation in the subset of GHG fluxes used for bootstrapping. This figure shows how much our carbon intensity estimates change when we use a subset with only tropical dams (which does not change our original estimates, with lines overlapping) and a set with all dams in the global dataset regardless of their climate zone (which increases our original estimates by ≈ 10%). This sensitivity analysis was run for the 100-year time horizon estimates.

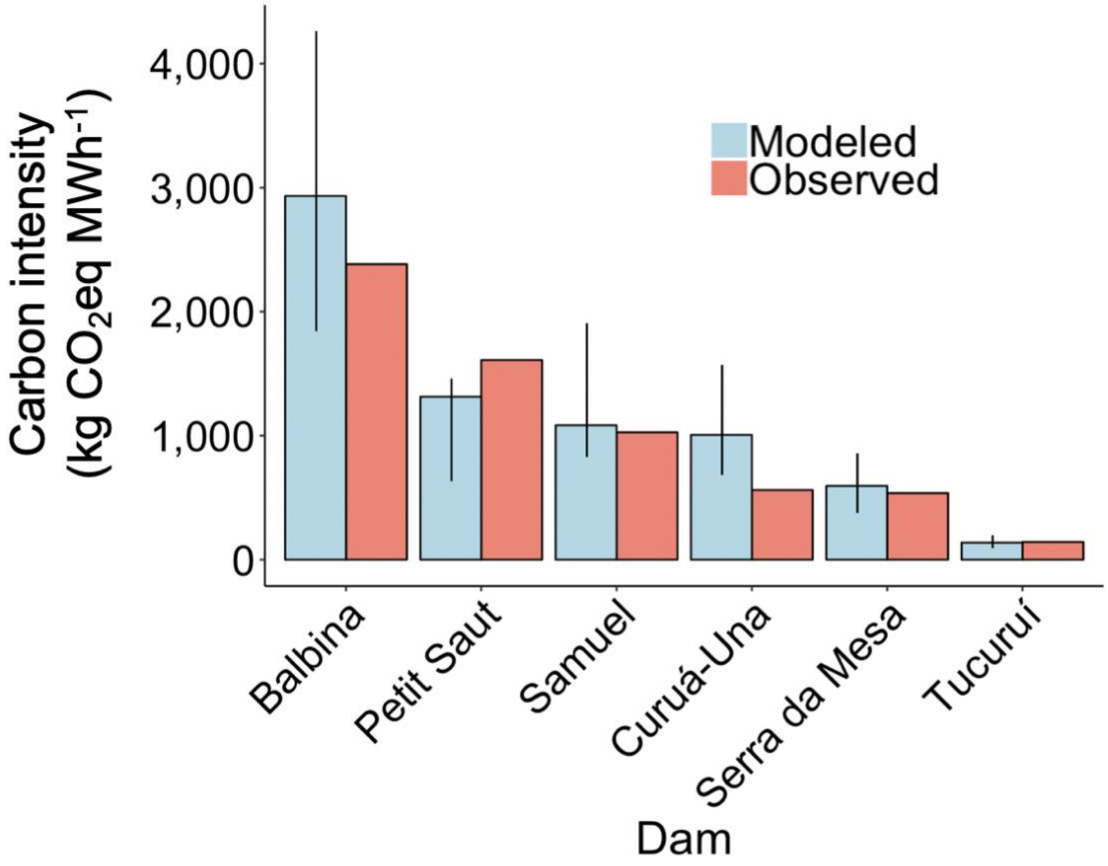

Supplementary Figure 7. Validation of calculated carbon intensities. We computed the carbon intensities with data for six existing dams in river basins contained within the Amazon biome limits. The blue bars indicate the average modeled carbon intensities and the error bars show the 95% confidence interval. The red bars indicate the observed carbon intensities. This analysis was run for a 100-year time horizon.

Supplementary Table 1. Total greenhouse gas (GHG) emissions of Amazon hydropower. Bootstrapped mean and 95% confidence intervals (CI) of estimated annual GHG emissions (in Tg CO_2eq yr^{-1}) of existing and proposed Amazon hydropower dams by region and country. For comparison, it is estimated that global reservoir surfaces (all types included, not only for hydropower) annually emit 800 Tg CO_2eq (95% CI: 500 – 1,200 Tg CO_2-eq)[12]. n = number of dams, GW = total installed capacity, in GW.

	Existing							
			20-year time horizon			100-year time horizon		
	n	GW	Bootstr. Mean	2.5% CI	97.5% CI	Bootstr. Mean	2.5% CI	97.5% CI
**Whole Amazon basin**	158	33	35	23	50	14	10	19
**Upland (> 500 m)**	91	9	1.4	1.0	1.9	1.3	1.2	1.5
**Lowland (< 500 m)**	67	24	33	21	47	13	9	17
**Bolivia**	27	1	0.32	0.22	0.52	0.19	0.16	0.23
**Brazil**	68	24	33	21	47	13	9	17
**Colombia**	0	0	-	-	-	-	-	-
**Ecuador**	27	5	0.3	0.2	0.4	0.5	0.5	0.6
**Peru**	36	3	0.8	0.5	1.0	0.5	0.4	0.6
	Proposed							
			20-year time horizon			100-year time horizon		
	n	GW	Bootstr. Mean	2.5% CI	97.5% CI	Bootstr. Mean	2.5% CI	97.5% CI
**Whole Amazon basin**	351	92	176	114	249	66	47	88
**Upland (> 500 m)**	126	25	10	6	14	5.4	4.4	6.4
**Lowland (< 500 m)**	225	67	166	107	234	60	42	81
**Bolivia**	16	11	29	19	41	10	7	14
**Brazil**	212	34	79	51	112	28	20	39
**Colombia**	2	0.7	0.3	0.2	0.5	0.18	0.14	0.22
**Ecuador**	36	11	0.5	0.4	0.7	1.2	1.1	1.2
**Peru**	85	35	67	43	94	24	17	33
